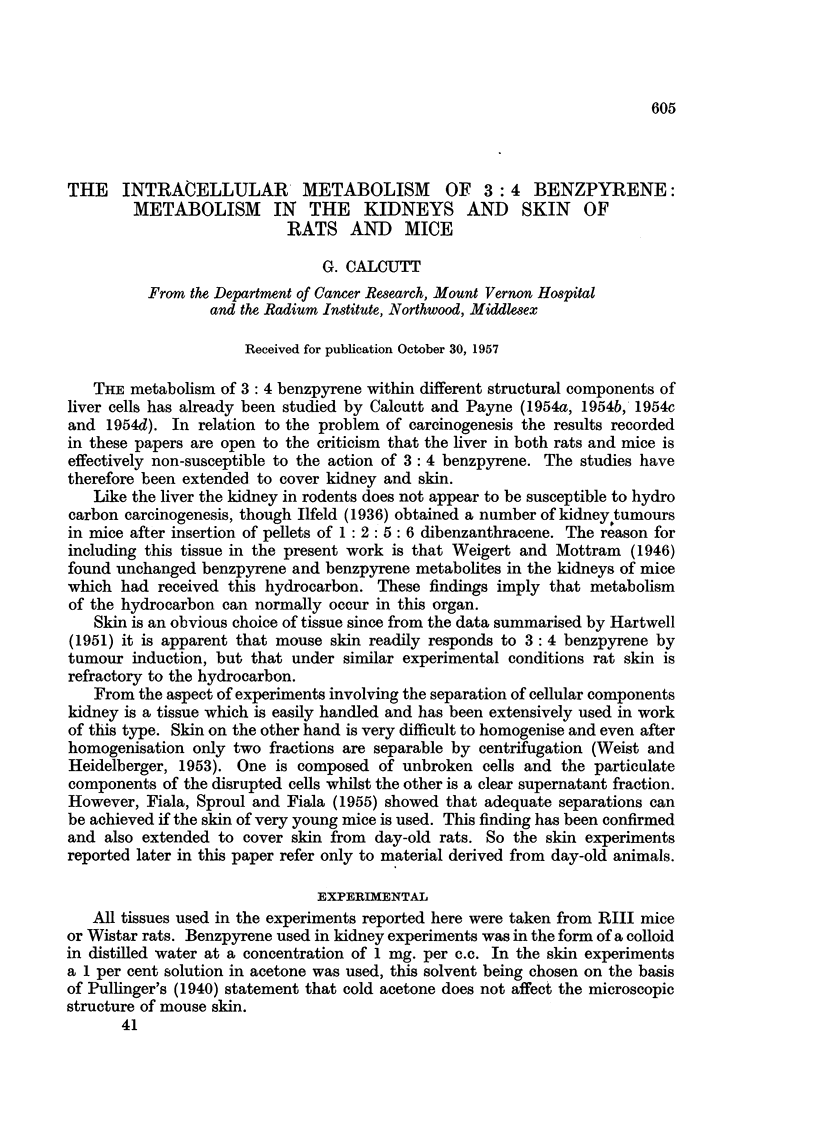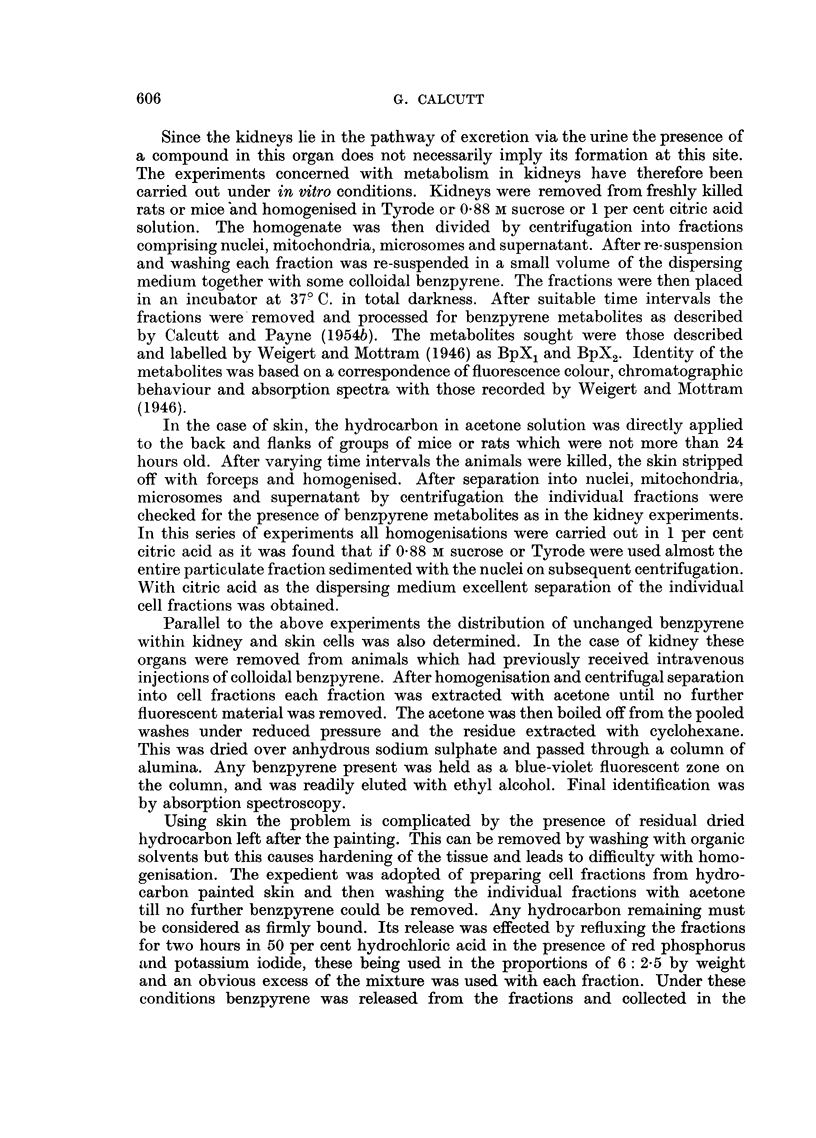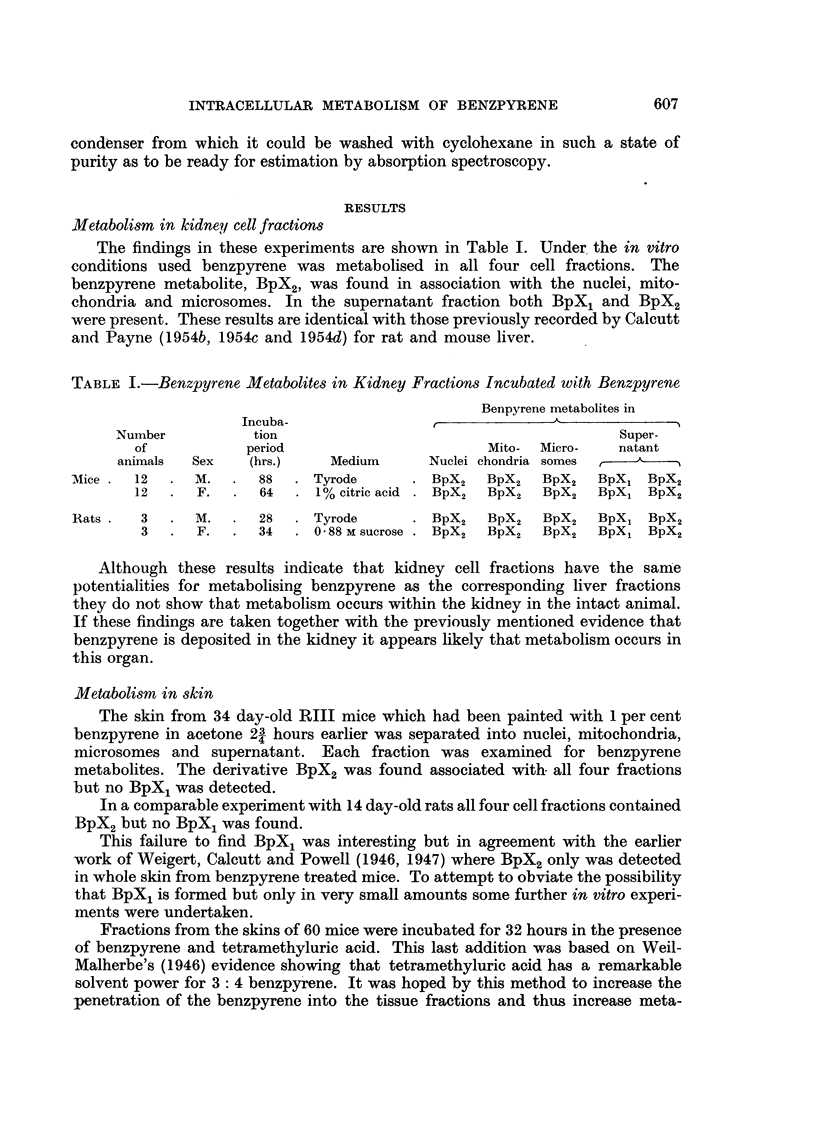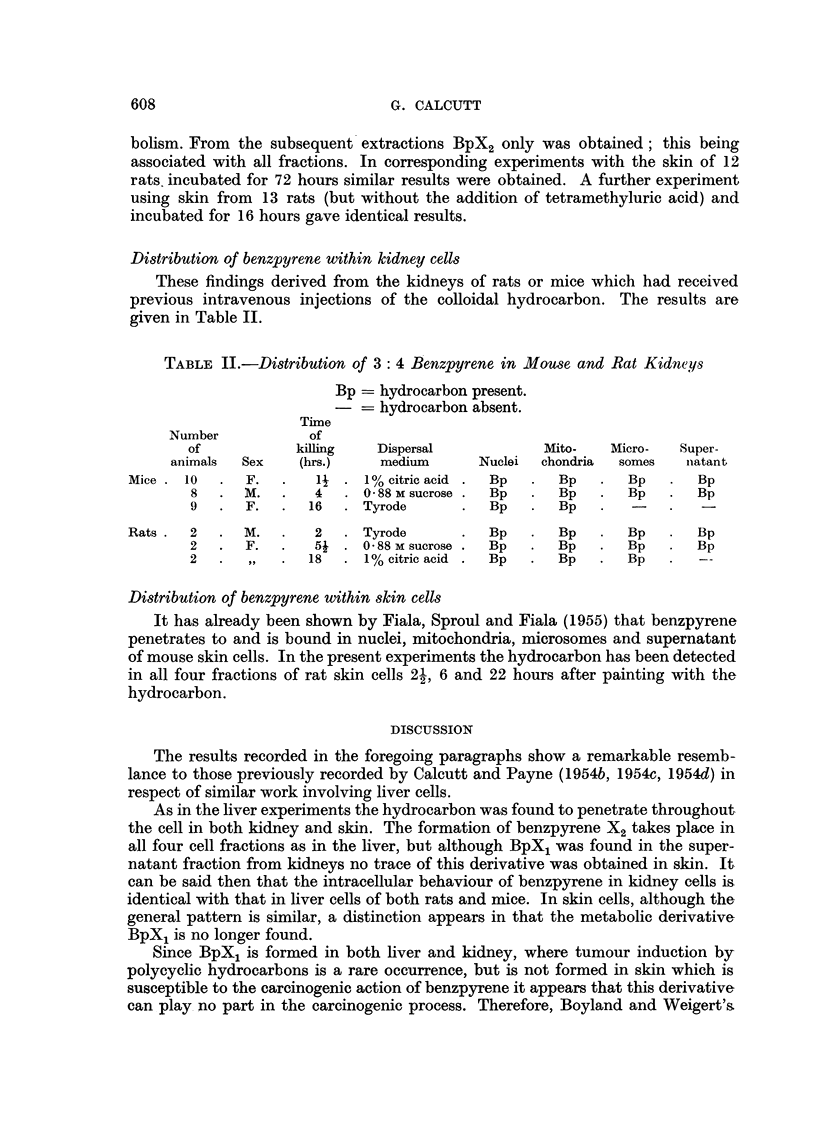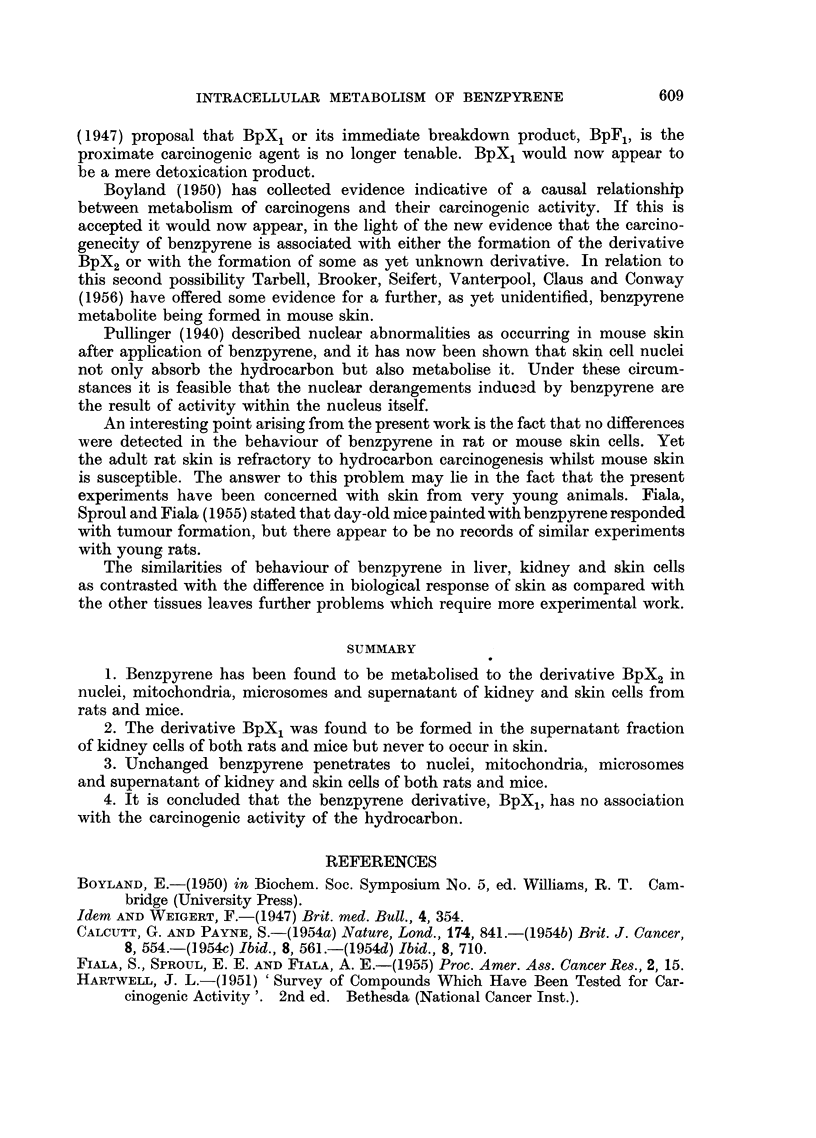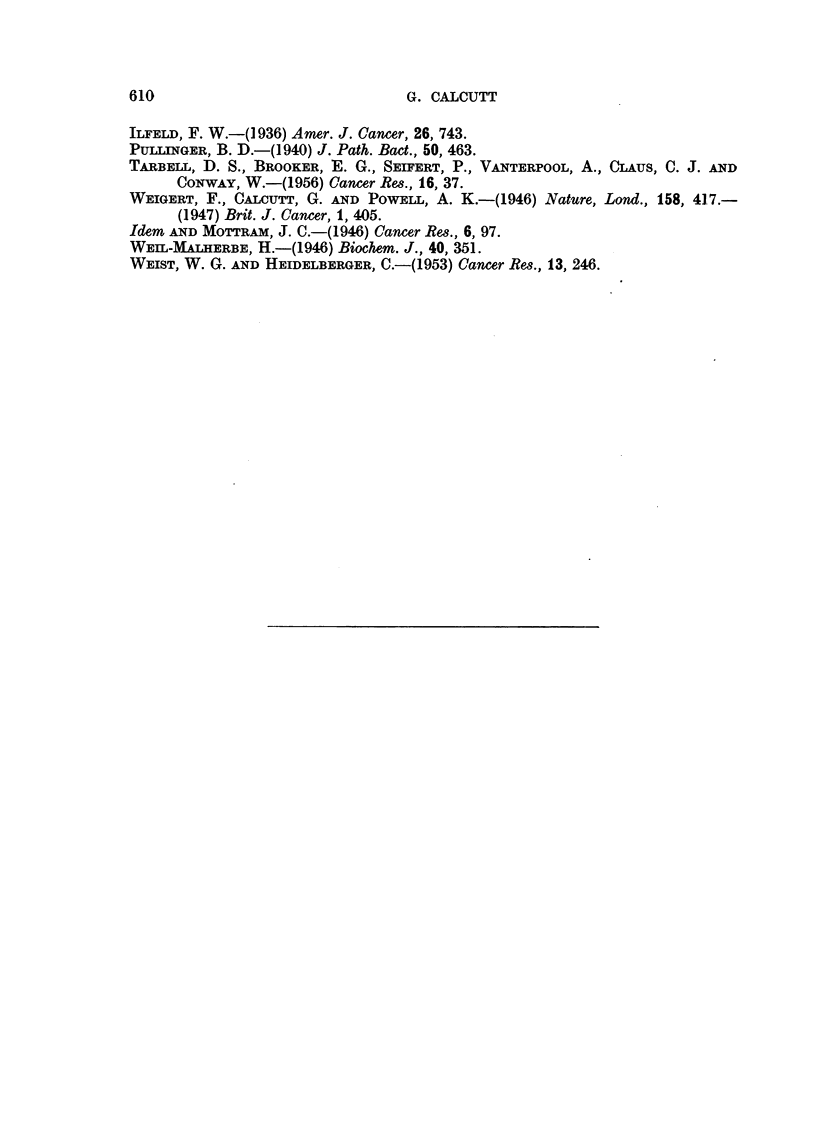# The Intracellular Metabolism of 3:4 Benzpyrene: Metabolism in the Kidneys and Skin of Rats and Mice

**DOI:** 10.1038/bjc.1957.74

**Published:** 1957-12

**Authors:** G. Calcutt


### THE INTRACELLULAR METABOLISM OF 3 : 4 BENZPYRENE: METABOLISM IN THE KIDNEYS AND SKIN OF RATS AND MICE

G. CALCUTT

*From the Department of Cancer Research, Mount Vernon Hospital and the Radium Institute, Northwood, Middlesex*

Received for publication October 30, 1957

THE metabolism of 3 : 4 benzpyrene within different structural components of liver cells has already been studied by Calcutt and Payne (1954*a*, 1954*b*, 1954*c* and 1954*d*). In relation to the problem of carcinogenesis the results recorded in these papers are open to the criticism that the liver in both rats and mice is effectively non-susceptible to the action of 3 : 4 benzpyrene. The studies have therefore been extended to cover kidney and skin.

Like the liver the kidney in rodents does not appear to be susceptible to hydro carbon carcinogenesis, though Ilfeld (1936) obtained a number of kidney tumours in mice after insertion of pellets of 1 : 2 : 5 : 6 dibenzanthracene. The reason for including this tissue in the present work is that Weigert and Mottram (1946) found unchanged benzpyrene and benzpyrene metabolites in the kidneys of mice which had received this hydrocarbon. These findings imply that metabolism of the hydrocarbon can normally occur in this organ.

Skin is an obvious choice of tissue since from the data summarised by Hartwell (1951) it is apparent that mouse skin readily responds to 3 : 4 benzpyrene by tumour induction, but that under similar experimental conditions rat skin is refractory to the hydrocarbon.

From the aspect of experiments involving the separation of cellular components kidney is a tissue which is easily handled and has been extensively used in work of this type. Skin on the other hand is very difficult to homogenise and even after homogenisation only two fractions are separable by centrifugation (Weist and Heidelberger, 1953). One is composed of unbroken cells and the particulate components of the disrupted cells whilst the other is a clear supernatant fraction. However, Fiala, Sproul and Fiala (1955) showed that adequate separations can be achieved if the skin of very young mice is used. This finding has been confirmed and also extended to cover skin from day-old rats. So the skin experiments reported later in this paper refer only to material derived from day-old animals.

### EXPERIMENTAL

All tissues used in the experiments reported here were taken from RIII mice or Wistar rats. Benzpyrene used in kidney experiments was in the form of a colloid in distilled water at a concentration of 1 mg. per c.c. In the skin experiments a 1 per cent solution in acetone was used, this solvent being chosen on the basis of Pullinger's (1940) statement that cold acetone does not affect the microscopic structure of mouse skin.

Since the kidneys lie in the pathway of excretion via the urine the presence of a compound in this organ does not necessarily imply its formation at this site. The experiments concerned with metabolism in kidneys have therefore been carried out under *in vitro* conditions. Kidneys were removed from freshly killed rats or mice and homogenised in Tyrode or 0·88 M sucrose or 1 per cent citric acid solution. The homogenate was then divided by centrifugation into fractions comprising nuclei, mitochondria, microsomes and supernatant. After re-suspension and washing each fraction was re-suspended in a small volume of the dispersing medium together with some colloidal benzpyrene. The fractions were then placed in an incubator at 37° C. in total darkness. After suitable time intervals the fractions were removed and processed for benzpyrene metabolites as described by Calcutt and Payne (1954*b*). The metabolites sought were those described and labelled by Weigert and Mottram (1946) as $BpX_1$ and $BpX_2$. Identity of the metabolites was based on a correspondence of fluorescence colour, chromatographic behaviour and absorption spectra with those recorded by Weigert and Mottram (1946).

In the case of skin, the hydrocarbon in acetone solution was directly applied to the back and flanks of groups of mice or rats which were not more than 24 hours old. After varying time intervals the animals were killed, the skin stripped off with forceps and homogenised. After separation into nuclei, mitochondria, microsomes and supernatant by centrifugation the individual fractions were checked for the presence of benzpyrene metabolites as in the kidney experiments. In this series of experiments all homogenisations were carried out in 1 per cent citric acid as it was found that if 0·88 M sucrose or Tyrode were used almost the entire particulate fraction sedimented with the nuclei on subsequent centrifugation. With citric acid as the dispersing medium excellent separation of the individual cell fractions was obtained.

Parallel to the above experiments the distribution of unchanged benzpyrene within kidney and skin cells was also determined. In the case of kidney these organs were removed from animals which had previously received intravenous injections of colloidal benzpyrene. After homogenisation and centrifugal separation into cell fractions each fraction was extracted with acetone until no further fluorescent material was removed. The acetone was then boiled off from the pooled washes under reduced pressure and the residue extracted with cyclohexane. This was dried over anhydrous sodium sulphate and passed through a column of alumina. Any benzpyrene present was held as a blue-violet fluorescent zone on the column, and was readily eluted with ethyl alcohol. Final identification was by absorption spectroscopy.

Using skin the problem is complicated by the presence of residual dried hydrocarbon left after the painting. This can be removed by washing with organic solvents but this causes hardening of the tissue and leads to difficulty with homogenisation. The expedient was adopted of preparing cell fractions from hydrocarbon painted skin and then washing the individual fractions with acetone till no further benzpyrene could be removed. Any hydrocarbon remaining must be considered as firmly bound. Its release was effected by refluxing the fractions for two hours in 50 per cent hydrochloric acid in the presence of red phosphorus and potassium iodide, these being used in the proportions of 6 : 2·5 by weight and an obvious excess of the mixture was used with each fraction. Under these conditions benzpyrene was released from the fractions and collected in the condenser from which it could be washed with cyclohexane in such a state of purity as to be ready for estimation by absorption spectroscopy.

### RESULTS

### *Metabolism in kidney cell fractions*

The findings in these experiments are shown in Table I. Under the *in vitro* conditions used benzpyrene was metabolised in all four cell fractions. The benzpyrene metabolite, $BpX_2$, was found in association with the nuclei, mitochondria and microsomes. In the supernatant fraction both $BpX_1$ and $BpX_2$ were present. These results are identical with those previously recorded by Calcutt and Payne (1954*b*, 1954*c* and 1954*d*) for rat and mouse liver.

TABLE I.—*Benzpyrene Metabolites in Kidney Fractions Incubated with Benzpyrene*

| | Number of animals | Sex | Incubation period (hrs.) | Medium | Benpyrene metabolites in | | | | |
|---|---|---|---|---|---|---|---|---|---|
| | | | | | Nuclei | Mitochondria | Microsomes | Supernatant | |
| Mice | 12 | M. | 88 | Tyrode | $BpX_2$ | $BpX_2$ | $BpX_2$ | $BpX_1$ | $BpX_2$ |
| | 12 | F. | 64 | 1% citric acid | $BpX_2$ | $BpX_2$ | $BpX_2$ | $BpX_1$ | $BpX_2$ |
| Rats | 3 | M. | 28 | Tyrode | $BpX_2$ | $BpX_2$ | $BpX_2$ | $BpX_1$ | $BpX_2$ |
| | 3 | F. | 34 | 0·88 M sucrose | $BpX_2$ | $BpX_2$ | $BpX_2$ | $BpX_1$ | $BpX_2$ |

Although these results indicate that kidney cell fractions have the same potentialities for metabolising benzpyrene as the corresponding liver fractions they do not show that metabolism occurs within the kidney in the intact animal. If these findings are taken together with the previously mentioned evidence that benzpyrene is deposited in the kidney it appears likely that metabolism occurs in this organ.

### *Metabolism in skin*

The skin from 34 day-old RIII mice which had been painted with 1 per cent benzpyrene in acetone $2\frac{3}{4}$ hours earlier was separated into nuclei, mitochondria, microsomes and supernatant. Each fraction was examined for benzpyrene metabolites. The derivative $BpX_2$ was found associated with all four fractions but no $BpX_1$ was detected.

In a comparable experiment with 14 day-old rats all four cell fractions contained $BpX_2$ but no $BpX_1$ was found.

This failure to find $BpX_1$ was interesting but in agreement with the earlier work of Weigert, Calcutt and Powell (1946, 1947) where $BpX_2$ only was detected in whole skin from benzpyrene treated mice. To attempt to obviate the possibility that $BpX_1$ is formed but only in very small amounts some further *in vitro* experiments were undertaken.

Fractions from the skins of 60 mice were incubated for 32 hours in the presence of benzpyrene and tetramethyluric acid. This last addition was based on Weil-Malherbe's (1946) evidence showing that tetramethyluric acid has a remarkable solvent power for 3 : 4 benzpyrene. It was hoped by this method to increase the penetration of the benzpyrene into the tissue fractions and thus increase metabolism. From the subsequent extractions $BpX_2$ only was obtained; this being associated with all fractions. In corresponding experiments with the skin of 12 rats incubated for 72 hours similar results were obtained. A further experiment using skin from 13 rats (but without the addition of tetramethyluric acid) and incubated for 16 hours gave identical results.

*Distribution of benzpyrene within kidney cells*

These findings derived from the kidneys of rats or mice which had received previous intravenous injections of the colloidal hydrocarbon. The results are given in Table II.

TABLE II.—*Distribution of 3 : 4 Benzpyrene in Mouse and Rat Kidneys*

Bp = hydrocarbon present.
— = hydrocarbon absent.

| | Number of animals | Sex | Time of killing (hrs.) | Dispersal medium | Nuclei | Mito-chondria | Micro-somes | Super-natant |
|---|---|---|---|---|---|---|---|---|
| Mice | 10 | F. | 1½ | 1% citric acid | Bp | Bp | Bp | Bp |
| | 8 | M. | 4 | 0·88 M sucrose | Bp | Bp | Bp | Bp |
| | 9 | F. | 16 | Tyrode | Bp | Bp | — | — |
| Rats | 2 | M. | 2 | Tyrode | Bp | Bp | Bp | Bp |
| | 2 | F. | 5½ | 0·88 M sucrose | Bp | Bp | Bp | Bp |
| | 2 | ,, | 18 | 1% citric acid | Bp | Bp | Bp | — |

*Distribution of benzpyrene within skin cells*

It has already been shown by Fiala, Sproul and Fiala (1955) that benzpyrene penetrates to and is bound in nuclei, mitochondria, microsomes and supernatant of mouse skin cells. In the present experiments the hydrocarbon has been detected in all four fractions of rat skin cells 2½, 6 and 22 hours after painting with the hydrocarbon.

### DISCUSSION

The results recorded in the foregoing paragraphs show a remarkable resemblance to those previously recorded by Calcutt and Payne (1954*b*, 1954*c*, 1954*d*) in respect of similar work involving liver cells.

As in the liver experiments the hydrocarbon was found to penetrate throughout the cell in both kidney and skin. The formation of benzpyrene $X_2$ takes place in all four cell fractions as in the liver, but although $BpX_1$ was found in the supernatant fraction from kidneys no trace of this derivative was obtained in skin. It can be said then that the intracellular behaviour of benzpyrene in kidney cells is identical with that in liver cells of both rats and mice. In skin cells, although the general pattern is similar, a distinction appears in that the metabolic derivative $BpX_1$ is no longer found.

Since $BpX_1$ is formed in both liver and kidney, where tumour induction by polycyclic hydrocarbons is a rare occurrence, but is not formed in skin which is susceptible to the carcinogenic action of benzpyrene it appears that this derivative can play no part in the carcinogenic process. Therefore, Boyland and Weigert's

(1947) proposal that $BpX_1$ or its immediate breakdown product, $BpF_1$, is the proximate carcinogenic agent is no longer tenable. $BpX_1$ would now appear to be a mere detoxication product.

Boyland (1950) has collected evidence indicative of a causal relationship between metabolism of carcinogens and their carcinogenic activity. If this is accepted it would now appear, in the light of the new evidence that the carcinogenecity of benzpyrene is associated with either the formation of the derivative $BpX_2$ or with the formation of some as yet unknown derivative. In relation to this second possibility Tarbell, Brooker, Seifert, Vanterpool, Claus and Conway (1956) have offered some evidence for a further, as yet unidentified, benzpyrene metabolite being formed in mouse skin.

Pullinger (1940) described nuclear abnormalities as occurring in mouse skin after application of benzpyrene, and it has now been shown that skin cell nuclei not only absorb the hydrocarbon but also metabolise it. Under these circumstances it is feasible that the nuclear derangements induced by benzpyrene are the result of activity within the nucleus itself.

An interesting point arising from the present work is the fact that no differences were detected in the behaviour of benzpyrene in rat or mouse skin cells. Yet the adult rat skin is refractory to hydrocarbon carcinogenesis whilst mouse skin is susceptible. The answer to this problem may lie in the fact that the present experiments have been concerned with skin from very young animals. Fiala, Sproul and Fiala (1955) stated that day-old mice painted with benzpyrene responded with tumour formation, but there appear to be no records of similar experiments with young rats.

The similarities of behaviour of benzpyrene in liver, kidney and skin cells as contrasted with the difference in biological response of skin as compared with the other tissues leaves further problems which require more experimental work.

### SUMMARY

1. Benzpyrene has been found to be metabolised to the derivative $BpX_2$ in nuclei, mitochondria, microsomes and supernatant of kidney and skin cells from rats and mice.
2. The derivative $BpX_1$ was found to be formed in the supernatant fraction of kidney cells of both rats and mice but never to occur in skin.
3. Unchanged benzpyrene penetrates to nuclei, mitochondria, microsomes and supernatant of kidney and skin cells of both rats and mice.
4. It is concluded that the benzpyrene derivative, $BpX_1$, has no association with the carcinogenic activity of the hydrocarbon.

### REFERENCES

BOYLAND, E.—(1950) *in* Biochem. Soc. Symposium No. 5, ed. Williams, R. T. Cambridge (University Press).

*Idem* AND WEIGERT, F.—(1947) *Brit. med. Bull.*, **4**, 354.

CALCUTT, G. AND PAYNE, S.—(1954*a*) *Nature, Lond.*, **174**, 841.—(1954*b*) *Brit. J. Cancer*, **8**, 554.—(1954*c*) *Ibid.*, **8**, 561.—(1954*d*) *Ibid.*, **8**, 710.

FIALA, S., SPROUL, E. E. AND FIALA, A. E.—(1955) *Proc. Amer. Ass. Cancer Res.*, **2**, 15.

HARTWELL, J. L.—(1951) 'Survey of Compounds Which Have Been Tested for Carcinogenic Activity'. 2nd ed. Bethesda (National Cancer Inst.).

ILFELD, F. W.—(1936) *Amer. J. Cancer*, **26**, 743.
PULLINGER, B. D.—(1940) *J. Path. Bact.*, **50**, 463.
TARBELL, D. S., BROOKER, E. G., SEIFERT, P., VANTERPOOL, A., CLAUS, C. J. AND CONWAY, W.—(1956) *Cancer Res.*, **16**, 37.
WEIGERT, F., CALCUTT, G. AND POWELL, A. K.—(1946) *Nature, Lond.*, **158**, 417.—(1947) *Brit. J. Cancer*, **1**, 405.
*Idem* AND MOTTRAM, J. C.—(1946) *Cancer Res.*, **6**, 97.
WEIL-MALHERBE, H.—(1946) *Biochem. J.*, **40**, 351.
WEIST, W. G. AND HEIDELBERGER, C.—(1953) *Cancer Res.*, **13**, 246.